# A Proposed Drought Response Equation Added to the Münch-Horwitz Theory of Phloem Transport

**DOI:** 10.3389/fpls.2020.505153

**Published:** 2020-11-04

**Authors:** John D. Goeschl, Lifeng Han

**Affiliations:** ^1^Department of Industrial and Systems Engineering, College of Engineering, Texas A&M University, College Station, TX, United States; ^2^School of Mathematical and Statistical Sciences, Arizona State University, Tempe, AZ, United States

**Keywords:** phloem transport, turgor pressure, drought stress, transport speed, concentration

## Abstract

Theoretical and experimental evidence for an effect of sieve tube turgor pressure on the mechanisms of phloem unloading near the root tips during moderate levels of drought stress is reviewed. An additional, simplified equation is proposed relating decreased turgor pressure to decreased rate kinetics of membrane bound transporters. The effect of such a mechanism would be to decrease phloem transport speed, but increase concentration and pressure, and thus prevent or delay negative pressure in the phloem. Experimental evidence shows this mechanism precedes and exceeds a reduction in stomatal conductance.

## Review of Theoretical Background

The Münch hypothesis of phloem transport has been expressed mathematically in various model forms by several authors (e.g., Goeschl et al., 1976; Thompson and Holbrook, 2003; Hölttä et al., 2006; Jensen et al., 2010; Payvendi et al., 2014). Mathematically consistent models must include equations for at least five dependent variables, (1) the rates of solute unloading [jU_i_] in sink areas, (2) osmotic influx and efflux [jW_i_] of water through the sieve tube membrane, (3) turgor pressure [P_i_], (4) transport speed (i.e., velocity along the sieve tube) [vS_i_], and (5) solute concentration [C_i_] along the sieve tube axis. An empirical equation (6) for a sixth variable, (6) viscosity of the phloem sap (ŋ_i_ assuming only sucrose at 25°C) was included in the model by Goeschl et al. (1976); from Swindells et al. (1958) to adjust the effective conductance to the phloem sap along the sieve tube [LS_i_]. The steady-state, algebraic form of these equations for each (ith) sieve element (or computational section) are as follows (see default values of the independent parameters in Table 1):

**TABLE 1:** Independent and input parameters used in Figure 1.

**Independent parameter**	**Symbol**	**Value**	**Units**
Membrane permeability	ξ	5 × 10^–5^	sec^–1^ cm
Leaf Apoplastic Water Potential	Ψ_(leaf)_	−0.4	MPa
Root Apoplastic Water Potential	Ψ _(root)_	−0.6	MPa
Gas Law Constant	R	8.3145	m^3^ Pa mol^–1^K^–1^
Reflection Coefficient	δ	1	
Radius of Sieve Tube	CELLRAD	1.2 × 10^–3^	cm
Length of Sieve Tube	LN	100	cm
Length of Sieve Elements	CELLN	0.02	cm
Length of Computational Section	SECLN	1	cm
Number of Loading Sections	NLSECS	5	
Number of Path Sections	NPSECS	85	
Number of Unloading Sections	NUSECS	10	
Hydraulic Conductance of Sieve Tube	LS	*	
Cross section area of S.T.**	AX	**	cm^2^
Area of sieve tube membrane***	AM	***	cm^2^
Total Vmax for each sink****	−Vmax_(tot)_	****	sec mol^–1^
Concentration for 1/2 vmax*****	Km	*****	molar

*Calculated for each computational section (Goeschl et al., 1976). **Calculated from ST radius = 1.2 m^–6^. ***Calculated from ST radius and the length of computational sections = 1 cm. ****Negative to indicate Unloading from the sieve tubes and distributed in a linear gradient along the Unloading Zone. *****Converted to mol cm^3^ in the model which uses CGS units.

(1) jW=iξ(Ψ-iP+iCRTi)δ

(2) vS=i-1Ls(P-i-1P)ii-1

(3) vSCi-1Axi-1+jL=ivSCiAxi

(4) vS(1-V¯C)i-1i-1Ax+jWAi=MivS(1-V¯C)ii-1Ax

(5) jU=i-VmaxCi/iKm+Ci

(6) ŋ=iExp(0.00531+(801.92C)iCi

Where ξ is membrane permeability, Ψ_i_ is apoplastic water potential, P_i_ is turgor pressure, δ is the membrane reflection coefficient, Ls_i_ is the hydraulic conductance of the Sieve Tube, Ax is the cross section area of the sieve tube, jL_i_ is the phloem loading rate, $\bar{V}$ is the partial molar volume of sucrose, A_M__i_ is the surface area of the sieve element, and Vmax_i_ and Km are the rate constants of the unloading transporter.

**FIGURE 1:**
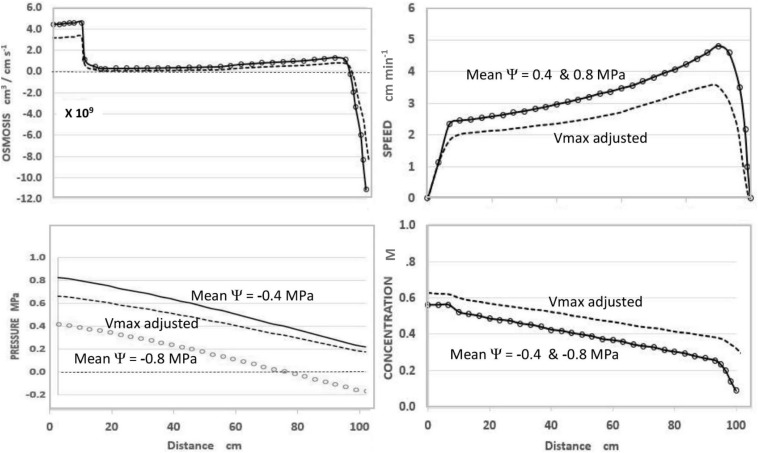
Effects of apoplastic water potential (Ψ) and effective unloading conductance (i.e., EUC assigned as Vmax of the Unloading enzyme system). **Solid Traces** MeanΨ = −0.4 MPa with Vmax = −1.72 ×10^–10^ mol sec^–1^. **Open Circles** MeanΨ = −0.8 MPa with Vmax = −1.72×10^–10^ mol sec ^–1^. **Dashed Traces** MeanΨ = −0.8 MPa with Vmax = −1.35 ×10^–10^ mol sec^–1^. Plotted from same data as Goeschl (2019, Figures 7–5) where the term **mPa** should have been **MPa**.

In the course of expressing various predictions of this steady-state model (Figure 1), it was observed that uniformly decreasing the value of apoplastic water potential (Ψ_i_) along the axis of a modeled sieve tube (i.e., maintaining the same slope or gradient of water potential) had no effect on the values or patterns of OSMOSIS (i.e., osmotic influxes and effluxes along the phloem axis), SPEED, or CONCENTRATION, but uniformly lowered the absolute value of PRESSURE (replotted using data from Goeschl, 2019).

As seen in Figure 1, and expected from the Münch hypothesis, the predicted turgor pressure always slopes downward from the loading zone to the unloading zone (in this case assumed to be along the root tips). At the chosen values of the independent parameters (Table 1) with a mean water potential of −0.4 MPa the predicted value of Turgor pressure is positive at all points (Figure 1, solid traces). If the mean water potential is lowered to −0.8 MPa, the predicted values of OSMOSIS, SPEED, and CONCENTRATION do not change (Figure 1, open circles, superimposed on solid traces). However, PRESSURE decreases uniformly along the axis (open circles) and reaches negative values approaching the terminal end of the sieve tube. Indeed, one could assign even lower mean values of water potential and the Pressure curve would reach negative values along the entire length of the sieve tube, but the other variables would be unaffected (i.e., open circles superimposed on the solid traces of Figure 1), so long as the slope of the pressure curve remained the same.

The question of whether phloem transport in real plants would continue to operate normally with negative pressures was raised by Lang (1974). Among the considerations is that the thin-walled sieve elements in the growing zone of roots are surrounded by newly formed parenchyma and other cells, known to maintain turgor pressures high enough to continue growth in moderate drought stress conditions (Hsiao and Acevedo, 1974; Sharp and Davies, 1985; Sharp et al., 1988; Hsiao and Xu, 2000; Hummel et al., 2010). It is likely that the sieve elements (possibly including the sieve plate pores) near the root tips would be compressed to smaller diameters, thus restricting the flow of phloem sap. There is also a possibility of plasmolysis under these conditions. The question then is whether plants have some mechanism to prevent or minimize the likelihood of negative pressures in the sieve tubes.

One hypothetical mechanism is a reduction in the Effective Unloading Conductance (EUC) of the sieve tubes in the root sinks, e.g., by virtue of a reduction of Vmax (Goeschl, 2019). This could occur in real plants as the sieve tube membranes are compressed, which may alter solute transporter and/or aquaporin protein configurations or expression levels, or membrane electro-potentials, and thus decrease the rate parameters of these mechanisms. This would be consistent with the known effects of turgor pressure on membrane transport kinetics (e.g., Hans et al., 1976; Patrick, 1994; Bell and Leigh, 1996; Geiger, 2011), on the distribution of photosynthates to various plant organs, especially roots (e.g., Hummel et al., 2010; Lemoine et al., 2013), and on related metabolism (Guo et al., 2018). Related changes in the xylem-phloem transport systems under reduced apoplastic water potential was predicted by the model of Hölttä et al. (2009). Reducing the EUC of the root sinks in a plant with more than one competitive sink would not necessarily decrease the amount of photosynthates unloaded into the growing roots, so long as the loading rate remained the same. In fact, phloem unloading in the root sinks could increase relative to other sinks (Hummel et al., 2010) since the relative change in sieve tube turgor would be greater in the roots, and total reduced EUC in all sinks would increase solute Concentration throughout the phloem network.

Using the model to predict the effect of reduced EUC in a plant with one hypothetical sink was accomplished by decreasing the collective Vmax_(tot)_ of the hypothetical Unloading Transporters from 1.72 × 10^–10^ mol sec^–1^ to 1.35 × 10^–10^ mol sec^–1^, while maintaining all other input parameters constant. As seen in Figure 1 (dashed traces), this resulted in a decrease in transport SPEED, an increase in CONCENTRATION (i.e., reciprocal effects), and substantially increased PRESSURE to be positive at all points. The relative change in pressure (along with Concentration) was proportionately greater near the root tips. In real plants this would presumably occur gradually and prevent pressure from becoming negative.

## Summary of Experimental Tests

**FIGURE 2:**
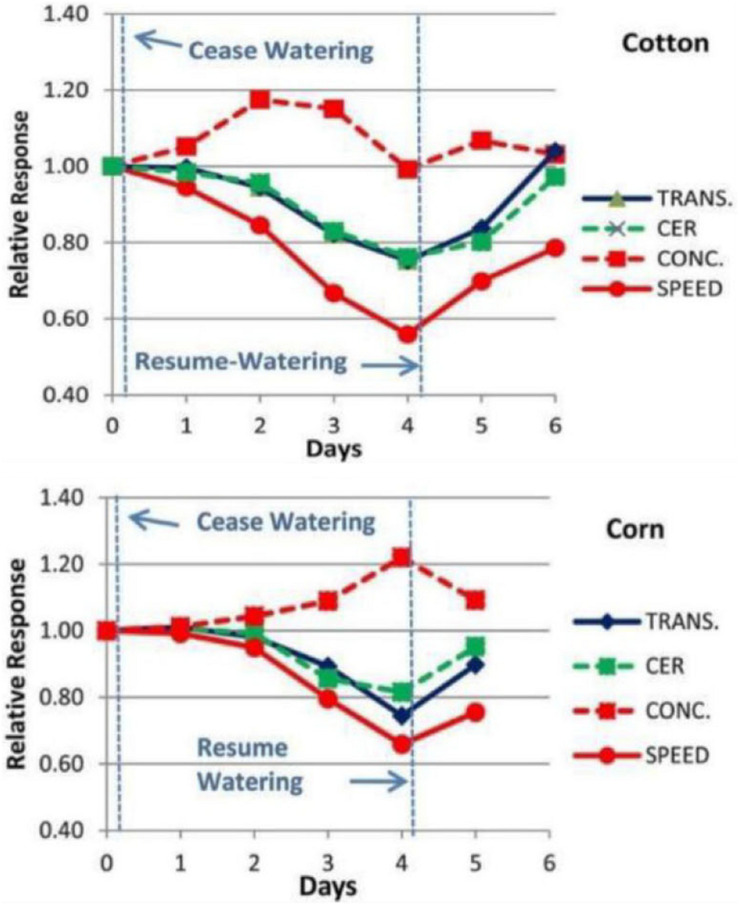
Example of the changes in Phloem Transport Speed and Concentration (measured experimentally by Carbor-11 Traces kinetic analysis, Goeschl et al., 1988; Goeschl, 2019) along with changes in CER and Transpiration of a Cotton and Corn plant during a 4 days “drydown” and re-watering experiment of the type conducted by Boyer (1970).

Experimental measurements were conducted using the Extended Square Wave Carbon-11 Tracer method (^11^C ESW, Fares et al., 1983; Goeschl et al., 1988) in individual, live, uninjured, undisturbed Cotton and Corn plants as they were subjected to decreasing water potentials over a period of 4 days (Figure 2, also see Goeschl, 2019, Figures 8-1 and 8-2). The importance of test plants being fully recovered from disturbance (in our case at least 48 h; Goeschl et al., 1988) or injury (in our case at least 72 h; Goeschl, 2019) is supported by similar comments by Patrick (1994). The results showed gradual decreases in transport speed and increased concentration, preceding by one or 2 days, and exceeding the amplitude of decreases in transpiration (TRANS) and Carbon Exchange Rate (CER, i.e., photosynthesis) by reduced stomatal conductance.

**FIGURE 3:**
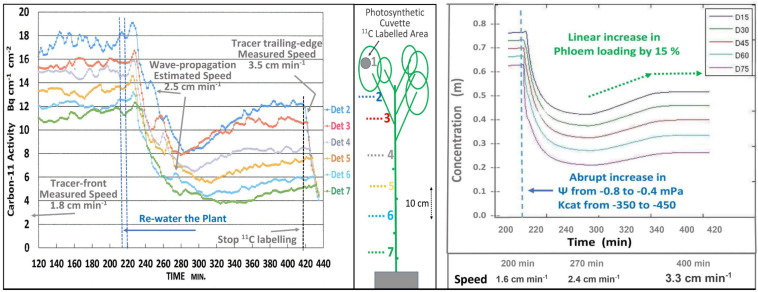
**Left**; Minute-by-minute ^11^C activities at numbered detector positions along a moderately drought-stressed Cotton plant during the steady state (final 300 min) of a 420 min Extended Square Wave input of ^11^CO_2_, before, during, and after re-watering of the plant. **Right**; Output of a time-dependent version of the Münch-Horwitz model of a phloem sieve tube subjected to the same set of input conditions and changes believed to have occurred in the experimental plant.

The short-term effects of re-watering (i.e., 4 min trickle irrigation of water at soil temperature) and the presumed restoration of high EUC on transport was seen on a minute-by-minute basis (Figure 3 Left). Since ^11^CO_2_ and ^12^CO_2_ were maintained at steady state equilibrium for 300 min, the downward trend is interpreted as simultaneous decreases in the concentrations of both tracer and trace. As illustrated in Figure 1, this results from the decreased EUC, not from dilution by increased apoplastic water potential. The 40 min decline reflects the capacitance effect of phloem volume. This was followed by an increase in photosynthesis, which led to an increase in both transport speed and concentration. Initial transport speed was measured by passage of the tracer front [at the beginning of the 120 min buildup phase (not shown)], by passage of the tracer trailing edge after 420 min, and by estimation from propagation velocity of the auto-induced wave near 260 min. The approximate 0.5x relation between wave velocity and transport speed is based on data from Goeschl et al. (1984), That wave, and the “noise” seen in these traces results from brief stoppages of phloem transport typical of Cotton (Goeschl et al., 1984).

The output of a time-dependent version of the phloem model (Figure 3, Right) programmed in MATLAB by LH based on Smith et al. (1980, see also Goeschl, 2019) closely matched the experimental results of Figure 3 Left.

## An Added Equation

Mathematical models at the beginning of their development, generally represent the simplest set of assumptions [Equations (1)–(6)]. Such a simple model may adequate to predict the results of the initial assumed circumstances. However, experimental tests may show it to be inadequate to express the effects of additional or changing circumstances. This appears to be the case for the Münch-Horwitz Theory under moderate drought stress conditions where the rate kinetics of the unloading mechanism decrease when the apoplastic water potential decreases (Figure 3).

If this proposed mechanism is true, and the rate kinetics of the unloading process are altered by the local turgor pressure at appropriate points along the sieve tubes, then the values of one or more of the Independent Parameters of the equation(s) representing the unloading mechanisms [in this hypothetical case the Michaelis–Menten Equation (5)] would become dependent variables as a function of pressure.

**FIGURE 4:**
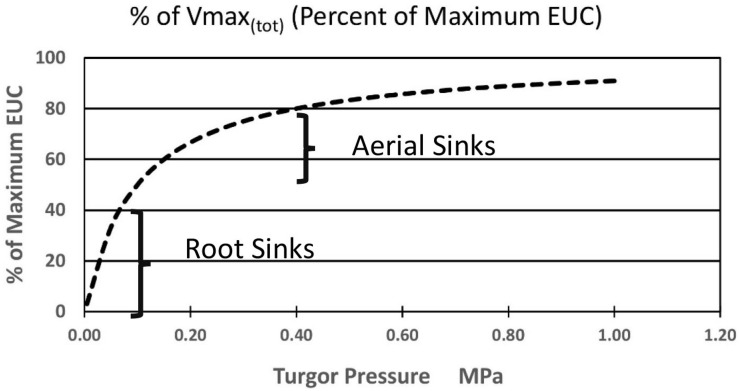
Hypothetical effect of turgor pressure on the value of Vmax_(tot)_ of the membrane-bound unloading enzyme system (i.e., transporters) in various metabolic sinks.

Again, starting with the simplest concepts and mathematics one can suggest the following added equation [Equation (7)] where the sum of the kinetic parameter Vmax_(__i__)_ of the membrane bound transporter in the root sink, or a system of enzyme activities, in the sinks (at the highest likely value of sieve tube turgor pressure of plants in a growth medium at Field Capacity moisture level) is altered as function of changing pressures. Note that this maximum value in some sinks may be set by a turgor homeostat mechanism as described by Hölttä et al., 2006, 2009). As a starting point, the following proposed formulation is based on the rationale that the transporter would be most sensitive as turgor pressure approached zero during moderate drought stress levels, and less sensitive as turgor pressure approached the levels of relatively unstressed plants. It would likely experience the greatest change near the root tips where the apoplastic water potential would undergo the greatest relative change. The term Kp is the pressure causing Vmax_(tot)i_ (in the ith computational section) to be 1/2 of its value at maximal apoplastic water potential and sieve tube pressure. A resulting plot is illustrated in Figure 4.

(7) Vmax=i-VmaxP(tot)⁢i/i(Kp+P)i

Obtaining realistic values for the parameters of any such equation would not be easy. Again, as a starting point, empirical measurements of values for phloem sap concentration and unloading rate in the root zone of real plants, needed to calculate EUC, could be accomplished by combining the ^11^C ESW tracer method (Fares et al., 1983; Goeschl et al., 1988) with recently developed “Micro-PET” imaging systems (e.g., Cherry et al., 1997; Beer et al., 2010; Weisenburger et al., 2012; Wang et al., 2014, and others). This could be performed by quantitative imaging of the final two or three cm length of a growing root, including the root tip as illustrated in Figure 11-4 of Goeschl (2019). Pressure could be measured by probes or estimated on the basis of sieve tube solute Concentration (calculated from measurements of ^11^C activity during last few minutes of an ^11^C ESW) and the measured values of plant apoplastic water potentials.

Relationships between other independent parameters, such as phloem loading rate, membrane permeability, and sieve tube diameter, in relation to dependent variables such as concentration and pressure may also exist in real plants and could result in additional equations.

Finally, progress toward a model of carbon flow through the entire plant might be approached by coupling a Phloem Transport model with mechanistic models of Photosynthesis (e.g., Zhu et al., 2007) and sink metabolism (e.g., Keener et al., 1979) to achieve the goals put forward by Demichele et al. (1978).

## Author Contributions

JG: models and experiments. LH: development of models. Both authors contributed to the article and approved the submitted version.

## Conflict of Interest

The authors declare that the research was conducted in the absence of any commercial or financial relationships that could be construed as a potential conflict of interest.
